# Tumor-Stromal Interactions Influence Radiation Sensitivity in Epithelial- versus Mesenchymal-Like Prostate Cancer Cells

**DOI:** 10.1155/2010/232831

**Published:** 2010-07-29

**Authors:** Sajni Josson, Starlette Sharp, Shian-Ying Sung, Peter A. S. Johnstone, Ritu Aneja, Ruoxiang Wang, Murali Gururajan, Timothy Turner, Leland W. K. Chung, Clayton Yates

**Affiliations:** ^1^Department of Urology, Emory School of Medicine, Atlanta, GA 30311, USA; ^2^Cedars-Sinai Medical Center, Los Angeles, CA 90048, USA; ^3^Department of Biology and Center for Cancer Research, Tuskegee University, Carver Research Foundation, Tuskegee, AL 36088, USA; ^4^Department of Radiation Oncology, Emory School of Medicine, Atlanta, GA 30322, USA; ^5^Department of Biology, Georgia State University, Atlanta, GA 30303, USA

## Abstract

HS-27a human bone stromal cells, in 2D or 3D coultures, induced cellular plasticity in human prostate cancer ARCaP_E_ and ARCaP_M_ cells in an EMT model. Cocultured ARCaP_E_ or ARCaP_M_ cells with HS-27a, developed increased colony forming capacity and growth advantage, with ARCaP_E_ exhibiting the most significant increases in presence of bone or prostate stroma cells. Prostate (Pt-N or Pt-C) or bone (HS-27a) stromal cells induced significant resistance to radiation treatment in ARCaP_E_ cells compared to ARCaP_M_ cells. However pretreatment with anti-E-cadherin antibody (SHEP8-7) or anti-alpha v integrin blocking antibody (CNT095) significantly decreased stromal cell-induced radiation resistance in both ARCaP_E_- and ARCaP_M_-cocultured cells. Taken together the data suggest that mesenchymal-like cancer cells reverting to epithelial-like cells in the bone microenvironment through interaction with bone marrow stromal cells and reexpress E-cadherin. These cell adhesion molecules such as E-cadherin and integrin alpha v in cancer cells induce cell survival signals and mediate resistance to cancer treatments such as radiation.

## 1. Introduction

Prostate cancer is the most frequent tumor in men, afflicting African American males to a greater degree than Caucasians. Morbidity and mortality are mainly attributable to metastasis; yet the mechanisms associated with progression are largely unknown. Localized carcinomas are readily removed surgically, but once a tumor has established metastases, current therapies are not curative and prolong survival by only a few years. Metastasis occurs through a multistep process, where metastatic cells must intravasate local tissues and enter into and survive in the blood stream. These cells then extravasate into the secondary tissue and initiate and maintain micrometastases at distant sites, with the end result being the development of a metastatic tumor [1, 2]. During each step of this process, cancer cells exhibit transdifferentiation properties that allow both the spatial and temporal expression of epithelial and mesenchymal properties in response to microenvironment signals and its own basic survival needs (e.g., motility and invasion versus proliferation). Thus, a model of cellular transitions, as opposed to a continual progression to permanent differentiation state, is emerging as a significant mechanism during metastasis. A greater understanding of these mechanisms will result in clinical improvements and a better control of the metastasis process.

Epithelial-mesenhymal transition (EMT) was first described during development [3, 4]; however an EMT-like phenotypic change has been observed in a number of solid tumors [5–7]. This transition is typically characterized by a loss in E-cadherin and cytokeratin expression. EMT in cancer, as in development, is associated with an increase in cell proliferation [8, 9] and the acquisition of a mesenchymal phenotype that includes vimentin, N-cadherin, and osteopontin expression. In both normal development EMT and cancer-associated EMT, the loss of E-cadherin is critical to the differentiation and maintenance of the epithelial phenotype and provides a structural link between adjacent cellular cytoskeletons, which is important for tissue architecture. Cells that have undergone EMT (E-cadherin negative mesenchymal cells) subsequently become more migratory and invasive and proceed to traverse underlying basement membranes, with an acquired ability to intravaste the surrounding local tissue and gain access to vascular conduits. As such, the loss of E-cadherin is rate limiting for EMT [10, 11]. Recent reports from this laboratory and others have described a mesenchymal to epithelial reverting transition (MErT) to occur, where mesenchymal-like prostate cancer cell lines reexpress E-cadherin to become epithelial-like, and reestablish cellular adhesion during colonization within the liver tumor microenvironment [12, 13]. These findings are shared in clinical metastases of various cancer origins including breast, colon, and bladder, where robust membrane expression of E-cadherin was observed, and the paired more differentiated primary tumors were E-cadherin negative [6, 14]. Thus, a reversion of the mesenchymal phenotype appears to be important in latter stages of metastasis.

Numerous studies have shown that the underlying influence of these cellular transitions is a consequence of tumor-stromal interactions [15, 16]. Coculture studies have found that the survival and proliferation of cancer cells are intimately linked to the soluble factors in the microenvironment, such as EGF, TGF-*β*, IGF-*l* that contribute to survival and the subsequent formation of macrometastasis [17–20]. However, these factors are not likely to have a direct effect during initial metastatic colonization, and thus heterotypic and homotypic cellular adhesion has been proposed to provide the necessary survival signals for successful colonization [21, 22]. Current state-of-the-art technology does not provide the necessary resolution to determine at the single cell level in patients or experimental *in vivo* systems, individual cells that have successfully colonized the secondary site. However, numerous reports have firmly established that cancer-stromal interactions *in vitro* or in three-dimensional (3D) assays accurately mimic the drug sensitivity/resistance behavior of those cells found within solid tumors *in vivo* in a preclinical or clinical setting [23]. Thus, we employed a novel coculture assay to determine the cellular plasticity of cancer cells promoted by the bone stroma and the effect of tumor-stromal interactions on irradiation therapy in prostate cancer.

The ARCaP model is the only robust prostate cancer bone metastatic model which demonstrates epithelial to mesenchymal transition(EMT). The ARCaP progression model consists of ARCaP_E_ (epithelial) and ARCaP_M_ (mesenchymal), where the ARCaP_E_ cells have a bone metastatic potential of 12.5% and the ARCaP_M_ cells have a bone metastatic potential of 100%. The ARCaP_E_ and ARCaP_M_ cells express the classical markers of EMT [24, 25]. Herein we present findings that ARCaP_M_ cells undergo MErT when cocultured within the bone microenvironment in 3D and 2D cultures. Additionally, ARCaP_E_ cells that retained an epithelial phenotype exhibited a measurable growth advantage and retained ability to form colonies, however only under coculture conditions with bone stroma. Furthermore, blocking the ability of ARCaP_E_ or ARCaP_M_ cells from E-cadherin-mediated cell-cell adhesion or integrin alpha v beta-associated adhesion significantly affected ARCaP cell survival within bone stroma and sensitized these cells to radiation treatment.

## 2. Methods

### 2.1. Cell Culture

The human prostate cancer cell lines, ARCAP_E_, ARCaP_M_, the HS-27a bone stromal cells (ATCC, Manasss, VA) and the Pt-N or Pt-C human prostate stromal cell. Isolation and characterization of the human prostate cancer RFP-ARCaP cell lines has been reported [26]. Red Fluorescent Protein- (RFP-) transfected cells were maintained in G418 (350 mg/mL) prior to experimentation. All cell lines were grown in a 5% CO_2_ incubator at 37°C in media consisting of T-medium (Invitrogen, Carlsbad, CA) supplemented with 5% (v/v) fetal bovine serum and 1% Penicillin-Streptomycin.

### 2.2. Cocultures

Initial cocultures were performed as previously described [12, 13] with modifications. Cocultures consisted of 50 000 cells/cm^2^ of HS-27a bone marrow stromal cells and 2000 cells/cm^2^ prostate cancer cells. Cocultures were maintained in serum-free T-media and plated on tissue culture dishes.

### 2.3. Clonogenic Assay

Cells were plated at low densities in six-well plates for 24 hours and then were irradiated with the appropriate radiation dose. Twenty-four hours later, the media were changed and cells were incubated until they formed colonies having at least 50 or more cells. Seventeen days later colonies were rinsed with PBS, stained with methanol/crystal violet dye, and counted. The colony formation ability was calculated as a ratio of the number of colonies formed, divided by the total number of cells plated, times the plating efficiency [(# of colonies formed ÷ total # cells plated) × plating efficiency]. For experiments with cocultures, cells were initially incubated on a mat of stromal cells for 24 hours and radiated; 4 hours later clonogenic assay was performed. For antibody-based experiments using anti-E-cadherin (15 *μ*g/mL, DECMA or SHEP8-7, Sigma) and anti-integrin alpha-v (20 *μ*g/mL, CNT095) antibody, cancer cells were treated with respective antibodies for 24 hours prior to plating them on a mat of stromal cells.

### 2.4. Radiation

External beam radiation was delivered on a 600 Varian linear accelerator (Varian Medical Systems, Inc.Palo Alto, CA) with a 6 MV photon beam. A 40 × 40 cm field size was utilized and Petri dishes were placed on 1.5 cm of superflab bolus. Monitor units (MUs) were calculated to deliver the dose to a depth of dmax at a dose rate of 600 MU/min.

### 2.5. Statistical Analysis

Representative findings are shown for all experiments, which were performed in triplicate, repeated a minimum of three times. Student's *t*-test was used to determine the statistical significance between groups.

## 3. Results

### 3.1. ARCaP EMT Model Undergoes a Mesenchymal-to-Epithelial Reverting Transition (MErT)

Recently, the ARCaP model has been described to closely mimic the patho-physiology of advanced clinical human prostate cancer bone metastasis [25]. The ARCaP_E_ cells were derived from single-cell dilutions of the ARCaP cells. These cells exhibit a cuboidal-shaped epithelial morphology with high expression of epithelial markers, such as cytokeratin 18 and E-cadherin. The lineage-derived ARCaP_M_ cells have a spindle-shaped mesenchymal morphology and phenotype. ARCaP_M_ cells have decreased expression of E-cadherin and cytokeratins 18 and 19 but increased expression of N-cadherin and vimentin. These cells have decreased cell adhesion and increased metastatic propensity to bone and adrenal glands [27]. The morphologic and phenotypic changes observed in the ARCaP_M_ cells closely resemble those of cells undergoing EMT. 

**Figure 1:**
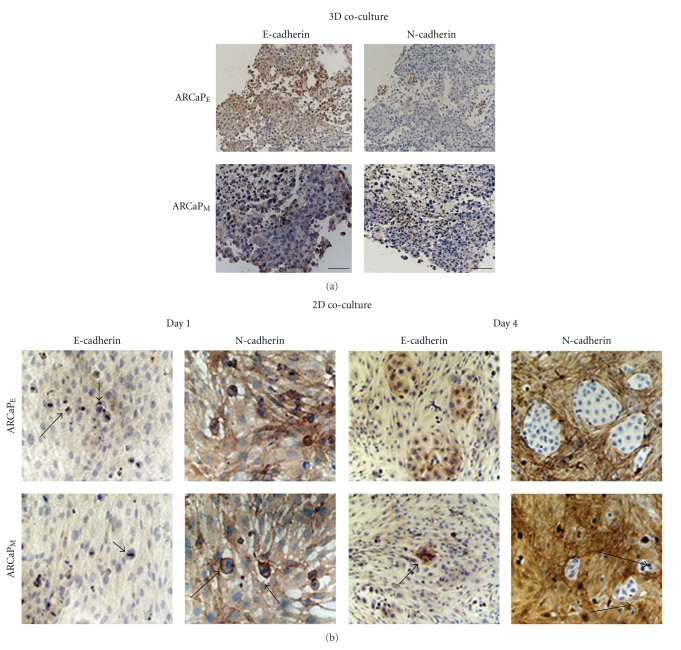
3D cocultures of ARCaP_E_ or ARCaP_M_ with HS-27a cells show E-cadherin expression. (a) 1 × 10^7^ ARCaP_E_ or ARCaP_M_ were cocultured with HS-27a cells in RWV for 3 days. Immunohistochemistry of organoids was stained with anti-E-cadherin or N-cadherin antibody. (b) 2D Cocultures of HS-27a were preformed utilizing a total of 50,000 cm^2^/HS-27a fibroblasts, after which 20,000 cm^2^ ARCaP_E_ or ARCaP_M_ were seeded on top of the fibroblast monolayer. The cocultures were maintained in serum-free medium for 1 or 4 days. Immunocytochemistry of cocultures over these time periods was performed utilizing anti-E-cadherin and N-cadherin antibodies. Shown are the EMT/MET of ARCaP_E_cells (top panels) and MErT of ARCaP_M_cells (bottom panels).

Previously, we have demonstrated a Mesenchymal to Epithelial *reverse *Transition (MErT) of metastatic prostate cancer cell lines within an experimental coculture model and confirmed in patients with liver metastasis [13, 28]. Our findings have recently been confirmed in prostate cancer bone metastasis where E-cadherin and *β*-catenin were robustly expressed in late stage carcinomas [29]. Therefore we sought to identify the significance of the bone microenvironment within the experimental ARCaP model. To assess cellular plasticity of the ARCaP EMT model, we coultured ARCaP cells with HS-27a cells in 3D RWV (rotary wall vessel) system for 3 days. ARCaP_E_ cells formed larger prostate organoids than ARCaP_M_ cells (data not shown). Upon immunohistochemical examination of organoids, we observed that both ARCaP_E_ and ARCaP_M_ express E-cadherin and lack N-cadherin expression (Figure 1(a)). To further examine the influence of tumor-stroma interactions over a multiday period we utilized a similar 2D cocultures method. Utilizing immunoctyochemical analysis, we observed a lack E-cadherin and robust N-cadherin staining after 1 day in both ARCAP_E_ and ARCaP_M_ cocultures. However by day 4, both ARCaP_E_ and ARCaP_M_ cells formed tumor nest that express E-cadherin and lack N-cadherin staining (Figure 1(b)). It is worthy to note that ARCaP_M_ tumor nest appeared to develop at much smaller extent, compared to ARCaP_E_ cocultures.

**Figure 2:**
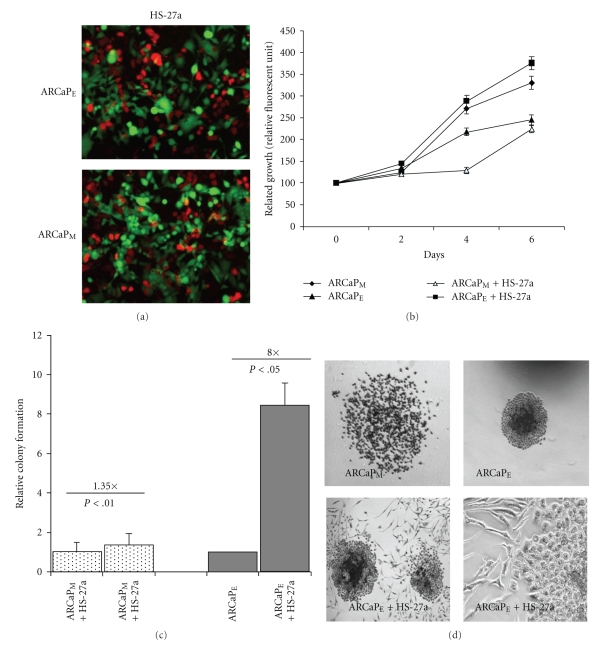
ARCaP_E_ cells show a growth and colony forming capacity advantage in presence of HS-27a cells. (a) and (b) ARCaP_M_ cells were cocultured in the presence of GFP-HS-27a cells over a 6-day period. Growth of RFP. ARCaP_E_ or ARCaP_M_ human prostate cancer cells was assessed by RFUs (relative fluorescent units) in the presence cocultures over a 6-day period. Results are means ± SE of three independent experiments. *P < .05 (students *t*-test) compared to cell number at day 1 ± SEM. (c) Clonogenic colony forming capacity of ARCaP_E_ and ARCaP_M_ prostate cancer cell after coculture ± SEM. ARCaP_M_ data were normalized to ARCaP_M_ control, and ARCaP_E_ data were normalized to ARCaP_E_ control (Note HS-27a induced slightly (1.35x) the growth of ARCaP_M_ cells but markedly (8x) the growth of ARCaP_E_ cells.). (d) ARCaP_E_ or ARCaP_M_ cells were cocultured with HS-27a cells. Shown are phase contrast images of colonies formed in the clonogenic assay.

 Since ARCaP_E_ cells formed larger tumor nest and spheroids when cocultured with HS-27a cells compared to ARCaP_M_ cells, we sought to further assess if HS-27a cells preferentially stimulated the growth of ARCaP_E_ cells versus ARCaP_M_ cells. Utilizing GFP-transfected HS-27a bone marrow stromal cells and RFP-transfected ARCaP_E_ or ARCaP_M_ cells (Figure 2(a)), we examined the proliferative ability of ARCaP cells in homotypic and coculture conditions. Growth of RFP-transfected ARCaP_E_ and ARCaP_M_ cells, respectively, was quantified by relative fluorescent units (RFU) of transfected cell lines over a 6-day period in homotypic cultures and coculture conditions (Figure 2(a)). As previously reported, homotypic cultured ARCaP_M_ shows significant growth compared to ARCaP_E_ homotypic cultures; however cocultures reversed this trend with ARCaP_E_ cells demonstrating the most significant growth (Figure 2(b)). We also confirmed these findings in ARCaP_E_ cells in coculture using clonogenic assay. Although ARCaP_M_ cells have a higher plating efficiency than ARCaP_E_ cells, ARCaP_E_ cells exhibited an 8-fold increase in their ability to form colonies after coculture compared to 1.35-fold increase of cocultured ARCaP_M_ cells (Figure 2(c)). Phase-contrast microscopy of colonies after coculture shows that ARCaP_M_ colonies appear loosely adherent, while ARCaP_E_ cells are compact and interact physically with few of the bone stromal fibroblast (Figure 2(d)). Taken together, these results demonstrate that ARCaP_M_ cells reexpress E-cadherin when grown with bone stromal cells for longer periods. Additionally, ARCaP_E_ cells which have high levels of E-cadherin gain enhanced growth and self-renewal ability when cocultured with bone stromal cells.

### 3.2. Stromal Cells Influence Radiation Treatment in Prostate Cancer Cells

**Figure 3:**
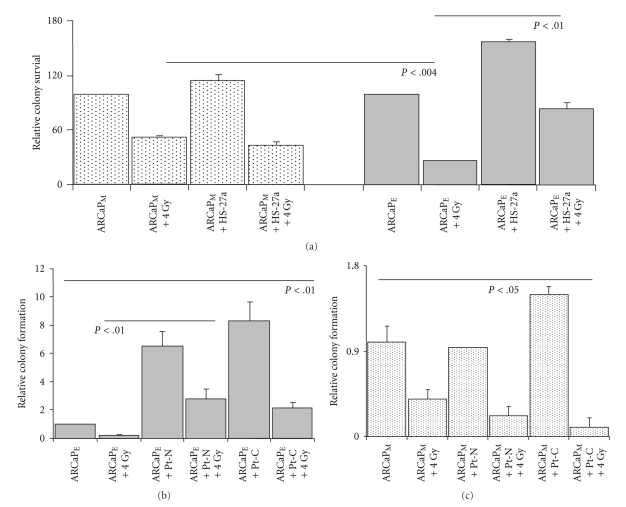
Cocultured ARCaP_E_ cells gain cell colony forming capacity and radiation resistance when grown with bone and prostate stromal cells. (a) ARCaP_E_ or ARCaP_M_ cocultured cells were irradiated 24 hours after coculture with HS-27a cells and cancer cell colony forming capacity was assayed using clonogenic assay. Results are means ± SE of three independent experiments. ARCaP_M_ experimental data are normalized to ARCaP_M_ control and ARCaP_E_ experimental data are normalized to ARCaP_E_ control (a). ARCaP_E_ cells cocultured with prostate stromal fibroblasts Pt-C (Cancer associated fibroblasts) or Pt-N (Normal/benign fibroblasts) were irradiated and compared to nonirradiated cocultures. Cell colony forming capacity was assayed by clonogenic assay. Data are normalized to ARCaP_E_ control levels. (b) ARCaP_M_ cells cocultured with Pt-C or Pt-N were irradiated and compared to nonirradiated cocultures (c). Cell colony forming capacity was assayed by clonogenic assay. Data are normalized to ARCaP_M_ control levels.

Mesenchymal cancer cells have been thought to be more tumorigenic, aggressive, and resistant to treatments when compared to epithelial cancer cells [30]. A similar trend was observed in both ARCaP_E_ and ARCaP_M_ cells after (4 Gy) irradiation treatment. ARCaP_M_ homotypic cancer cells are more resistant to radiation treatment compared to ARCaP_E_ homotypic cancer cells (Figure 3(a)). However, ARCaP_M_ cocultures did not affect the radiation sensitivity of ARCaP_M_ cancer cells. The highly sensitive ARCaP_E_ cells exhibit a significant increased resistance to radiation therapy, up to 3-fold, as result of their interaction with bone stromal cells (Figure 3(a), *P* < .01). 

To further assess the role of the prostate stromal cells on tumor-stromal interactions influencing ARCaP cellular behavior, we cocultured paired prostate stromal fibroblasts isolated either from normal (Pt-N) or from cancer-associated regions (Pt-C) [31]. Again, ARCaP_E_ cells cocultured with (Pt-N) or (Pt-C) exhibited a 7-fold and 8-fold increase in colony formation, respectively (Figure 3(b), *P* < .01). We also saw a similar trend in a growth analysis assay (data not shown). However when measuring clonogenic ability after radiation treatment, ARCaP_E_ cells cocultured with either Pt-N or Pt-C had increased radiation resistance, with a 2-fold difference observed between homotypic cultured cells. Although a significant increase in clonogenic formation was observed in Pt-C versus Pt-N cocultures (*P* < .05), this did not significantly effect the radiation sensitivity of ARCaP_M_ cells (Figure 3(c)). Taken together, both bone and prostate stromal cell have a grown inductive effect on ARCaP_E_ cancer cells and mediate radiation resistance (up to 2-3 fold) in epithelial cancer phenotype, but not in ARCaP_M_ mesenchymal cancer cells.

### 3.3. Blocking Adhesive Contact Effects Radiation Sensitivity of Cocultured ARCaP Cells

**Figure 4:**
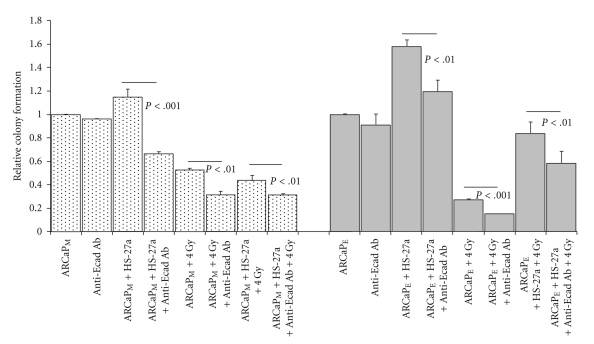
Effect of Anti-E-cadherin antibody on tumor-stroma interactions. A. ARCaP_M_ and ARCaP_E_, cells were pretreated with Anti-E-cadherin antibody (SHEP8-7), cocultured with HS-27a stromal cells for 24 hours, and radiated with 4 Gy. Cell colony forming capacity was assayed using clonogenic assay. ARCaP_M_ data are normalized to ARCaP_M_ control levels, and ARCaP_E_ data are normalized to ARCaP_E_ control levels.

The importance of cell adhesion (i.e., cell-cell and cell-ECM adhesion) on the survival of disseminated cancer cells has been well documented as a requirement for colonization and survival within the metastatic microenvironment [32–34]. Therefore we utilized a well-known E-cadherin blocking antibody (SHEP8-7) and a pan-integrin antibody (CNT095) that targets human alpha-v-integrin and also was shown to block prostate tumor growth within bone [35]. Since ARCaP_E_ cells express high levels of the epithelial marker E-cadherin, and ARCaP_M_ cells can be microenvironmentally induced to express E-cadherin, we tested whether either of these blocking antibodies would affect the colony forming ability of either ARCaP_E_ or ARCaP_M_ bone stroma-cocultured cells. Pretreatment with E-cadherin antibody did not affect the colony forming capacity of either ARCaP_E_ or ARCaP_M_ homotypic cultured cells; however it significantly reduced the ability of ARCaP_M_-  (*P* < .001) and ARCaP_E_-  (*P* < .01) coultured cells to form colonies (Figure 4). Additionally, E-cadherin blocking antibody pretreatments further increased sensitivity to radiation treatment of ARCaP_M_ cells in homotypic and cocultured conditions, similarly (*P* < .01). E-cadherin blocking antibody-pretreated ARCaP_E_ cells showed the most significant increased sensitivity to radiation treatment in homotypic compared cocultured conditions (*P* < .001), however a significant reduction in colony formation, to a lesser extent, was observed in ARCaP_E_ cocultured cells (Figure 4, *P* < .01). Therefore, targeting E-cadherin limited both epithelial and mesenchymal cells ability to form colonies after coculture with bone stromal cells.

**Figure 5:**
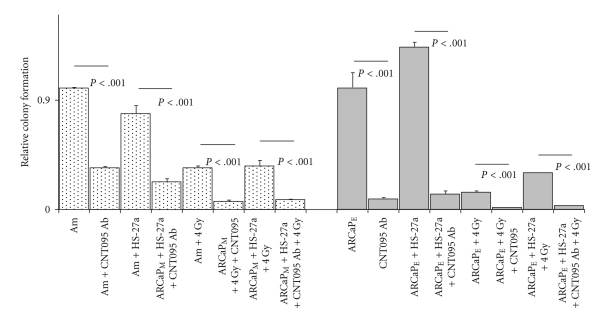
Effect of Anti-alpha v integrin (CNT095) on tumor-stroma interactions. ARCaP_M_ and ARCaP_E_, cells were pretreated with CNT095 antibody was cocultured with HS-27a stromal cells for 24 hours, and radiated with 4 Gy. Cell colony forming capacity was assayed using clonogenic assay. ARCaP_M_ data are normalized to ARCaP_M_ control levels, and ARCaP_E_ data are normalized to ARCaP_E_ control levels.

To determine the influence of intergin alpha v cell adhesion with bone microenvironment, we performed similar clonogenic formation assay. Pretreatment with CNT095 antibody significantly decreased the clonogenic ability of both ARCaP_M_ and ARCaP_E_ cells in homotyic cultures (Figure 5, *P* < .001). Additionally, CNT095 significantly decreased bone stroma-induced radiation resistance in cancer cells in both ARCaP_M_  (*P* < .001) and ARCaP_E_  (*P* < .001) cancer cells, with the most significant reduction in cocultured conditions (*P* < .001) (Figure 5). Taken together, these results suggest that bone stroma-induced radiation resistance is mediated through both E-cadherin and integrin alpha v beta signaling in epithelial and mesenchymal cells. Thus, E-cadherin and integrin alpha v beta appear to present novel targets for metastatic and radiation resistant cells.

## 4. Discussion

It is well documented in prostate and others cancers that EMT is associated with initial transformation from encapsulated to invasive carcinomas. The mesenchymal phenotype, which is required for dissemination, has been suggested to revert to an epithelial phenotype in distant metastasis [13, 14, 29, 36]. This has been evidenced in the primary tumors which lack E-cadherin expression and, showing nuclear *β*-catenin expression, show strong membrane staining for both E-cadherin and *β*-catenin in metastatic liver [13] or bone microenvironment [28, 29]. We have previously shown, in commonly utilized prostate cancer cells lines DU-145 and PC-3, that reexpression of E-cadherin and reversion of the mesenchymal phenotype is a rate limiting for metastatic seeding of primary rat hepatocytes [13]. Since bone metastasis is most prevalent in prostate cancers, we sought to extent these finding utilizing the ARCaP model, which is the first prostate cancer EMT model demonstrating histomorphological features and classical markers in a lineage-derived series of cells, to determine the functional relationship of this cellular transition. Whether this is accomplished through exposure to soluble growth factors or the bone microenvironment, the end result decreased differentiation with increased metastatic potential [25, 27, 37]. 

Our initial results show that ARCaP_M_ cells maintained in 3D Rotary Wall Vessel (RWV) or 2D cocultures underwent MErT when cocultured with HS-27a bone stromal cells, as shown through expression of E-cadherin and of N-cadherin expression (Figures 1(a) and 1(b)). Moreover ARCaP_E_ cells show a significant enhancement in colony formation (8×) and significant growth pattern comparable to ARCaP_M_ (1.35×) cocultures (Figures 2(b) and 2(c)). A recent report has shown through RFP cell tracking that selected ARCaP_E_ clones after *in vivo* inoculation into the bone microenvironment gives rise to both ARCaP_E_ and ARCaP_M_ populations [37]. These findings coupled with our observed reversion of ARCaP_M_ cells to ARCAP_E_ like cells suggest that tumor-stromal-induced cellular plasticity gives rise to distinct populations of cancer cells within bone microenvironment, the mesenchymal phenotype and its kinetic characteristics (motility/invasive), and the epithelial characteristics necessary for secondary tumor development. The fact that the ARCaP_M_ cells have an increase propensity for metastasis compared to ARCaP_E_ cells suggest that dissemination from the primary tumor mass requires the mesenchymal phenotype. However a mesenchymal to epithelial transition is associated with initial metastatic seeding and subsequent formation of a cohesive tumor mass within the bone microenvironment. This hypothesis is supported in a bladder cancer model, where lineage-derived series of EMT-transformed mesenchymal-like cells exhibit increased lung metastasis *in vivo*; however secondary tumor formation is predominantly enhanced by the presence of epithelial cells compared to mesenchymal cells [38]. 

Since epithelial reversion enhances the growth of tumor cells in bone microenvironment, and this is observed in multiple experimental models and clinical metastases, there is a question of whether this transition is required for metastatic seeding and therefore an avenue for therapeutic intervention(s). To gain insight into the importance of this reversion, we utilized ionizing radiation on ARCaP_E_ and ARCaP_M_ homotypic and cocultured cells. Our results show that ARCaP_E_ homotypic cultures when compared to ARCaP_M_ homotypic cultures are more sensitive to radiation treatment (Figure 3(a)). However in the presence of bone or prostate stromal cells, ARCaP_E_ cells gained increased radiation resistance, with increased proliferative and colony forming capacity (Figures 2(b) and 2(c)). This phenomenon was not observed in the ARCaP_M_ cocultures. To determine the underlining causes of this observation, we hypothesized that cell-cell interactions through E-cadherin or cell-ECM interactions through integrins may mediate the stromal induced proliferative effect and radiation resistance in ARCaP_E_ cancer cells. Using E-cadherin neutralizing antibody (SHEP8-7) and pan-anti-integrin alpha v antibody (CNT095), we were able to significantly block the stromal induced colony forming ability on ARCaP_E_ cancer cells (Figures 4 and 5). Additionally, both antibodies significantly blocked the radiation resistance of ARCaP_E_ in cocultured conditions (Figures 4 and 5). The E-cadherin neutralizing antibody also had an effect on homotypic ARCaP_M_-radiated cells and ARCaP_M_ cells within cocultures (Figure 4). Thus it appears that blocking bone stroma-induced reexpression of E-cadherin in ARCaP_M_ in the presence of bone stromal cells reduced the colony forming capacity of these cells (Figure 4). The decreased radiation sensitivity of E-cadherin expressing cells compared to cells lacking E-cadherin expression has recently been demonstrated in a cocultured model of MCF-7 (E-cad positive) and MDA-MB-231 (E-cad negative) cells with normal and radiation-induced senescent fibroblast [39], where radiation in MCF-7 cells showed enhanced resistance to radiation treatment compared to MDA-MB-231 cells. These findings are consent with our model of a reepithelization requirement within tumor microenvironment. 

CNT095 antibody was toxic to both ARCaP_M_ and ARCaP_E_ homotypic and cocultured cells. Additionally CNT095 increased radiation sensitivity, even to a greater extent than E-cadherin neutralizing antibody treatment (Figure 5). These findings are consistent with our results of CTN095 treatment that causes a significantly reduced number of tumors generated by C4-2B cells, along with a concomitant increase of cortical bone in mice (unpublished data). Although C4-2B cells have not been observed to undergo EMT, this would suggest that targeting the cell-ECM *in vitro* and* in vivo* could be limiting the cell cohesiveness necessary for metastatic tumor formation. 

Targeting of cell adhesion as a therapeutic approach has been proposed previously. E-cadherin neutralizing antibody (SHEP8-7) has been shown to sensitize multicellular spheroids to microtubule binding therapies in the taxane family in HT29 human colorectal adenocarcinoma cells [23]. A more recent observation is that survival of androgen receptor-expressing differentiated prostate cells is dependent on E-cadherin and PI3K, but not on androgen, AR, or MAPK [40]. Given the predominate role for PI3K in cell survival and reports that PI3K is rapidly recruited to cell membrane to stabilize E-cadherin junctions [40] and that PI3K activation requires integrin alpha v activity [41] suggests that PI3K is possibly responsible for the increased growth and colony formation gained within the tumor microenvironment. Thus in the absence of stimulating growth factors, it is possible that E-cadherin/PI3K or integrin alpha v/PI3K is involved in a signaling cascade that is initiated by the tumor microenvironment, at least during initial metastatic seeding. 

In conclusion, our data demonstrate that the E-cadherin and integrin alpha nctional adhesive interaction is a possible adjuvant therapy avenue for patients treated with radiation. Although an in-depth *in vivo* exploration of targeting epithelial-like versus mesenchymal-like cells is necessary to translate these findings to the clinical situation, our results indeed raise critical questions as to how we view prostate cancer metastasis and subsequently target metastatic tumor cells for therapy. Additionally, we have generated an *in vitro* model, that closely mimics the clinical situation, to delineate in a stepwise manner the dynamic tumor-host interaction(s) that promote cellular plasticity in the later stages of metastasis. The identification of further key molecules driving MErT in this system holds promise for the development of preventative and therapeutic strategies to minimize metastatic disease.
